# A Method to Improve the Characteristics of EPDM Rubber Based Eco-Composites with Electron Beam

**DOI:** 10.3390/polym12010215

**Published:** 2020-01-15

**Authors:** Gabriela Craciun, Elena Manaila, Daniel Ighigeanu, Maria Daniela Stelescu

**Affiliations:** 1 National Institute for Laser, Plasma and Radiation Physics, # 409 Atomistilor St., 077125 Magurele, Romania; gabriela.craciun@inflpr.ro (G.C.); daniel.ighigeanu@inflpr.ro (D.I.); 2 National R&D Institute for Textile and Leather—Leather and Footwear Research Institute, # 93 Ion Minulescu St., 031215 Bucharest, Romania; dmstelescu@yahoo.com

**Keywords:** EPDM rubber, wood sawdust, electron beam irradiation, dibenzoyl peroxide, cross-liking, physico-chemical characteristics

## Abstract

A natural fiber reinforced composite, belonging to the class of eco composites, based on ethylene-propylene-terpolymer rubber (EPDM) and wood wastes were obtained by electron beam irradiation at 75, 150, 300, and 600 kGy in atmospheric conditions and at room temperature using a linear accelerator of 5.5 MeV. The sawdust (S), in amounts of 5 and 15 phr, respectively, was used to act as a natural filler for the improvement of physical and chemical characteristics. The cross-linking effects were evaluated through sol-gel analysis, mechanical tests, and Fourier Transform Infrared FTIR spectroscopy comparatively with the classic method with dibenzoyl peroxide (P) applied on the same types of samples at high temperature. Gel fraction exhibits values over 98% but, in the case of P cross-linking, is necessary to add more sawdust (15 phr) to obtain the same results as in the case of electron beam (EB) cross-linking (5 phr/300 kGy). Even if the EB cross-linking and sawdust addition have a reinforcement effect on EPDM rubber, the medium irradiation dose of 300 kGy looks to be a limit to which or from which the properties of the composite are improved or deteriorated. The absorption behavior of the eco-composites was studied through water uptake tests.

## 1. Introduction

Ethylene-propylene-diene monomer (EPDM) rubber is a versatile polymer containing low compound costs. The stability of its saturated backbone structure determines the manifestation of a good resistance at heat and oxidation and also at ozone or weather ageing [1]. Its very good physical and chemical properties make it extremely suitable for obtaining automotive parts, sports goods, packaging materials, etc. [2]. For vulcanizing rubber compounds, many curing systems have been developed: sulfur, peroxides, metal oxides, phenolic resins, quinines. Of these, the first two were the most used for cross-linking of rubber materials until recently [3]. Even if the sulfur vulcanization has been known and applied for over 150 years, the complex chemistry of sulfur vulcanization is still not clearly understood. Both free radical and ionic mechanisms are considered as chemical pathways [3,4,5]. Both unsaturated and saturated elastomers can be cross-linked by means of organic peroxides but, for the second type, the sulfur curing systems cannot be applied [3]. Currently, the rubber processing using high energy radiations is a method increasingly used for designing new materials based on the modification of polymers [1,6,7]. By the use of gamma rays or electron beams C–C bonds, as in the case of peroxide cross-linking, are formed [1]. Particularly, the electron beam irradiation has many advantages over the mentioned curing systems such as high degrees of cross-linking and extremely strong bonds, which are obtained directly by C–C linkage. The process occurs at room temperature so the degradation generated by temperature is avoided. The curing cycles are shorter than in classical treatments and the productivity is higher, which is very suitable for both thin and thick products (depending on the electrons’ energy and the density of the product to be irradiated) and, lastly, a very important aspect of the process is that it does not generate material wastes [8,9,10,11]. Due to the radiation ability to initiate chemical reactions at any temperature, under any pressure and in any phase (gas, liquid, or solid) without the use of catalysts, very reactive intermediates are formed [12]. These intermediates can follow several reaction paths, which result in rearrangements and/or the formation of new bonds. Therefore, radiation offers a productive way of forming polymer bridges to bond together very different polymeric and non-polymeric elements of an engineering structure [12]. The fully saturated bonds in the main chain with a lack of quaternary carbon atoms make EPDM rubber suitable for radiation cross-linking that can induce additional cross-linking and/or scission of the polymeric chain [1]. The obtained product characteristics depend on the content and nature of fillers or additives added but also on dose and dose rates applied [1,8,13,14]. The oxidation degradation phenomena that can occur during processing must be taken into account and a method to retard or even to suppress the oxidative degradation is compounded with antioxidants, like Irganox, Tinuvin, or N-phenyl-N’-isopropyl-p-phenylenediamine IPPD [15]. Antioxidants are autoxidation inhibitors, which interfere in the free radical reactions that take place during processing and/or irradiation process. This leads to the incorporation of oxygen into the rubber molecules forming hydroperoxides that feed the chain reaction with new radicals [15,16,17]. The resistance to thermo-oxidative degradation of irradiated EPDM rubber is greatly improved by the addition of antioxidants [15,18,19,20]. However, the radiation curing differs from thermal curing, which is carried out at ambient temperature under closely controlled conditions, such as radiation dose, dose rate, penetration depth. This form of curing ultimately results in a more well-defined end product [21].

The EPDM use in so many different applications is due to the capacity to accept large amounts of fillers as silica or carbon black that can significantly improve its properties [1,7,22]. However, the concern regarding the demonstrated adverse effects on occupational health (silicosis, tuberculosis, cancer, autoimmune diseases, etc. [23,24,25] due to the use of the mentioned reinforcing fillers made as natural fibers are now under attention to replace them [7,26,27,28]. In addition, the growing global environmental and social concern, and new environmental regulations have forced the search for new composites and green materials, compatible with the environment. For these reasons but also from others related to energy saving, favorable processing properties, dimensional stability, and not least biodegradability potential, the natural fiber reinforced composites were called eco-composites [29] and the wood sawdust was taken under study as a possible active filler substitute [30,31,32] initially in classical methods of cure consisting of repeated heating cycles in hot presses [8]. The filler amount, particles dimension, and process characteristics as an irradiation dose and dose rate are important for the obtained product properties especially due to the poor interfacial adhesion between the polymeric matrix and hydrophilic lingo-cellulosic fillers observed in classical treatments [8,33,34].

The goal of the paper is to comparatively present the cross-linking effects induced by two different methods of cure, electron beam irradiation, and dibenzoyl peroxide in order to obtain a polymeric eco-composite based on EPDM rubber and wood sawdust. The influence of filler loading and irradiation dose on cross-linking was studied through physical and chemical investigations as sol-gel analysis, mechanical tests, and Fourier Transform Infrared (FTIR) spectroscopy. The absorption behavior of the eco-composites was studied through water uptake tests.

## 2. Materials and Methods

### 2.1. Materials and Sample Preparation 

The raw materials that were used in the experiments were as follows: (I) Ethylene-propylene-diene terpolymers (EPDM) rubber as eco-composite matrix was of Nordel 4760 type, produced by Dow Chemical Company (Michigan, MI, USA) (mooney viscosity of 70 ML_1+4_ at 120 °C, ethylene content of 70%, 5-ethylidenenorbornene (ENB) content of 4.9 wt %, density of 0.88 g/cm^3^ and crystallinity degree of 10%), (II) Polyethylene glycol (PEG) as process aid was of PEG 4000 type supplied by Advance Petrochemicals Ltd. (Ahmedabad, India) (density of 1128 g/cm^3^ and melting point in the range of 4–8 °C), (III) Pentaerythritol tetrakis(3-(3,5-di-tert-butyl-4-hydroxyphenyl)propionate) as an antioxidant was of Irganox 1010 type bought from BASF Schweiz (Basel, Switzerland), (IV) Dibenzoyl peroxide as a cross-linking agent was of Perkadox 14-40B type from AkzoNobel Chemicals (Deventer, The Netherlands) (density 1.60 g/cm^3^, 3.8% active oxygen content, 40% peroxide content, pH 7), (V) sawdust was of fir wood type obtained from a local sawmill in Romania (Sebes, Romania) (size particles—mash 250–270, single type of wood).

Blends were prepared on an electrically-heated laboratory roller. For preparation of the polymeric composites, the blend constituents were added in the following sequences and amounts: 100 parts of EPDM were rolled until binding for 1–2 min, than 3 phr of PEG 4000, and 1 phr Irganox 1010 were added and embedded for another 3–4 min and finally 5 and 15 phr of wood sawdust were added and mixed for 2–4 min until the homogenization. Blends were removed from the roll in the form of the sheet that is about 2 mm thick. Test specimens were obtained by compression molding at 160 °C and a pressure of 150 MPa using an electrical press for 5 min. Plates were then cooled to room temperature under pressure. Process variables were as follows: temperature between 25–50 ± 5 °C, friction 1:1.1, and total blending time 8–14 min. Plates required for physical and mechanical tests with sizes of 150 × 150 × 2 mm^3^ were obtained by pressing in a hydraulic press at 110 ± 5 °C and 150 MPa [8].

Samples vulcanized with dibenzoyl peroxide were prepared in the same way as those for the electron beam, while adding 8 phr of the vulcanizing agent dibenzoyl peroxide Perkadox 14-4B in a hydraulic press at 160 °C for 20 min.

### 2.2. Experimental Installation and Sample Irradiation

Samples obtained as above and packed in polyethylene film for minimizing the oxidation were irradiated at 75, 150, 300, and 600 kGy in atmospheric conditions and at room temperature of 25 °C using the ALID-7 electron beam accelerator from National Institute for Laser, Plasma and Radiation Physics, Magurele, Romania. The nominal values of the electron beam (EB) parameters were as follows: energy of 5.5 MeV, peak current of 26 mA, output power of 134 W, and 3.75 μs pulse repetition frequency of 50 Hz [8]. 

The irradiation process performance depends on the rigorous control of the irradiation dose and dose rate [35,36]. In our experiments, the process dose rate was of 3.5 kGy/min. The primary standard graphite calorimeter was used for radiation dosimetry. In order to assure the equality between the entry and the exit irradiation dose of the irradiated samples, but also for an efficient use of the electron beam, the penetration depth was calculated according with the following equation [8,36].

(1) E=2.6⋅t⋅ρ+0.3

where *E* (MeV) is the electron beam energy, *t* (cm) is the sample thickness, and *ρ* (g·cm^−3^) is the sample density (in our case, 1 g·cm^−3^). 

The proper thickness of samples subjected to EB irradiation was calculated as being of 20 mm [8,37].

### 2.3. Laboratory Tests

Laboratory tests were carried out on EPDM samples with and without sawdust cross-linked by EB irradiation and by dibenzoyl peroxide. The sample codes are as follows: (1) EPDM-EB for samples without sawdust cross-linked by electron beam irradiation, (2) EPDM-EB-S 5 and EPDM-EB-S 15 for samples containing 5 and 15 phr of sawdust cross-linked by EB irradiation, (3) EPDM-P for samples without sawdust cross-linked with dibenzoyl peroxide, (4) EPDM-P-S 5 and EPDM-P-S 15 for samples containing 5 and 15 phr of sawdust cross-linked using dibenzoyl peroxide.

#### 2.3.1. Mechanical Characteristics

The mechanical properties of samples were evaluated using specific and proper equipment and instruments in accordance with the international standards in force as follows: a Schopper tensile tester according to ISO 37/2017 for tensile strength, a hardness tester according to ISO 7619-1/2011 for hardness, and a Schob test instrument according to ISO 4662/2017 for elasticity [8,11,37].

#### 2.3.2. Cross-Linking Evaluation

Sol-gel analysis and cross-link density determination were carried out on EPDM-EB, EPDM-EB-S, EPDM-P, and EPDM-P-S samples as in our previous works [8,11,37]. In order to determine the cross-linked products, gel content (gel fraction) was used as the solvent (toluene) extraction method [8,11,37]. The samples cross-link density (*ν*) was determined on the basis of equilibrium solvent-swelling measurements in toluene by applying the modified Flory-Rehner equation for tetra functional networks. The Flory-Huggins polymer-solvent interaction term $\chi_{12}$ for the EPDM-toluene system was of 0.49 [8,38,39].

#### 2.3.3. Fourier Transform Infrared Spectroscopy (FTIR)

The structure of the EPDM-EB-S and EPDM-P-S composites cross-linked by EB irradiation and dibenzoyl peroxide were analyzed by FTIR measurements using TENSOR 27 spectrophotometer (Bruker, Germany). The absorption spectra were obtained as 30 scans mediation, in the range of 4000–600 cm^−1^, with a resolution of 4 cm^−1^ [8].

#### 2.3.4. Water Uptake Evaluation

The water absorption in EPDM-EB-S and EPDM-P-S was evaluated as in our previous work [8], by immersion in distilled water until they no longer absorb water, in accordance with ISO 20344/2011. 

#### 2.3.5. Rubber-Filler Interaction

The interaction between the EPDM rubber and filler (wood sawdust) was also analyzed as in our previous work [8] using the Kraus theory that helps assess an interfacial interaction in filler-reinforced rubber composites [40,41,42]. 

## 3. Results and Discussion

### 3.1. Mechanical Characteristics

By EB irradiation, cross-linking and chain scission can occur. The first one appears frequently at lower irradiation doses, up to 150 kGy, while the second one is associated with the breaking of C–C bonds at higher doses [37,43,44]. Due to the aliphatic chain, which has low resistance to ionizing radiation action, the degradation can be predominant as compared to cross-linking in EPDM rubber at higher irradiation doses [37,44,45].

The mechanical properties of the EPDM/EPDM-S composites cross-linked by peroxide (P) and electron beam (EB) are presented comparatively.

**Figure 1:**
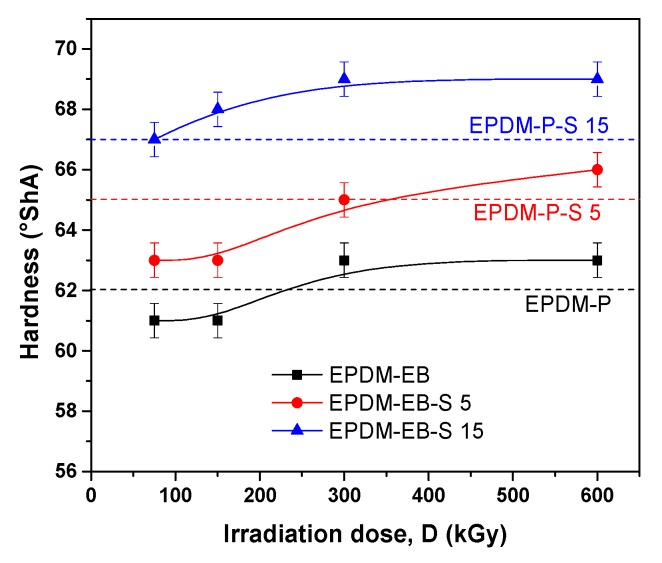
The influence of sawdust amount and the cross-linking method (P/EB) on composite hardness.

As shown in Figure 1, hardness was improved by the addition of sawdust (S) in the case of P cross-linking and by the irradiation dose increasing and S addition, in the case of EB cross-linking. The S addition increased hardness with 8% in the case of P cross-linking and with 13% in the case of EB cross-linking. An irradiation dose up to 300 kGy appear to be sufficient to obtain the reinforcement effect and to the extent of cross-linking in the polymeric material [1,8,46]. 

**Figure 2:**
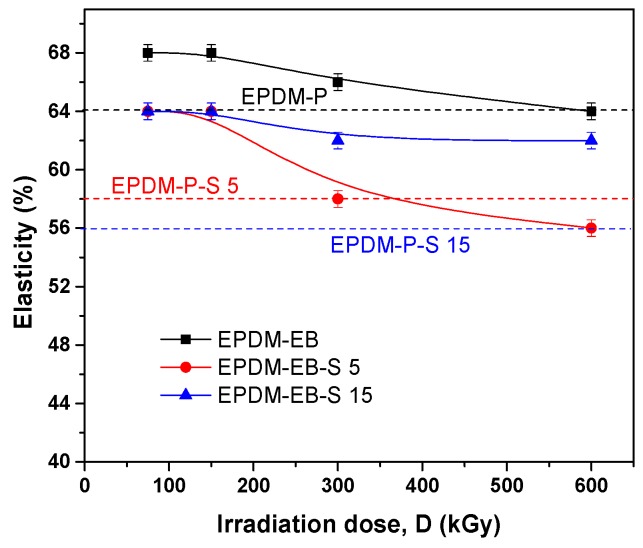
The influence of sawdust amount and cross-linking method (P/EB) on composites elasticity.

In Figure 2, the composites’ elasticity behavior is presented. In the cases of both cross-linking methods, the addition of S led to the elasticity decreasing against the samples without S. However, it should be noted that the EB cross-linking method, with or without S, is at least as effective as P cross-linking at a low irradiation dose of 150 kGy and more effective until the irradiation dose of 300 kGy. After that, it can be seen that only the samples containing 15 ppm of S and cross-linked by EB are still more elastic than samples containing 5 and 15 ppm of S and cross-liked by P. As in the case of hardness, low and middle irradiation doses appear to be sufficient to maintain the elasticity decreasing below 6%. If, in the case of P cross-linking is obvious, the presence of S reduces the degree of elasticity and segment mobility of the cured composites. In the case of EB cross-linking, the addition of S does not diminish the strain energy [8,46,47] and does not increase the composite hysteretic behavior [8,47,48].

**Figure 3:**
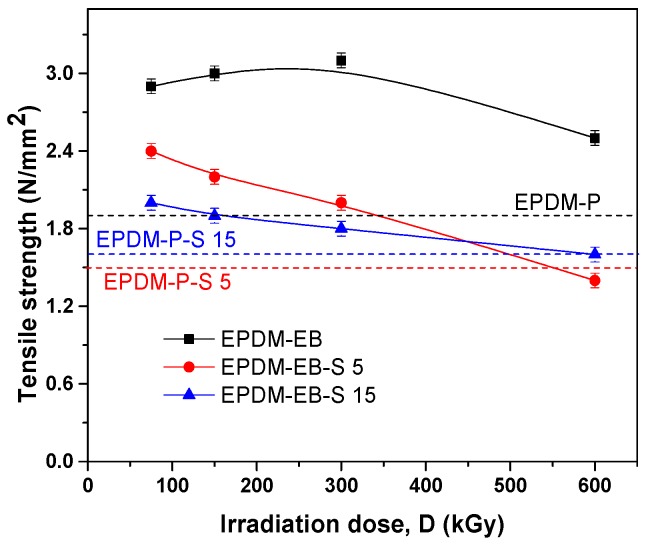
The influence of sawdust amount and cross-linking method (P/EB) on composites’ tensile strength.

The composites’ tensile strength behavior is presented in Figure 3. It is easy to observe the superiority of the EB cross-linking method comparatively with the P cross-linking method in the case of EPDM blends, irrespective of the irradiation dose. 

In the case of P cross-linking, the addition of 5 ppm of S decreased the tensile strength with 20% while the addition of 15 ppm of S decreased the tensile strength with only 10%. In the case of EB cross-linking, at low and medium irradiation doses (up to 150 kGy), the addition of 15 ppm of S maintain the tensile strength over the P cross-linking values, even if comparatively with the values without S addition. The tensile strength decreased between 25% and 35% [1].

The results can conduct to the idea of a not-so-strong interaction between the rubber and filler or an unsatisfactory adhesion of the filler in the polymeric matrix, which is insufficient to constrain the motion of the chains [8,49,50]. It looks like the loading with low S amounts and irradiation with doses up to 300 kGy can be a solution to maintain the composites’ properties similar with those obtained in the case of P cross-linking. 

**Figure 4:**
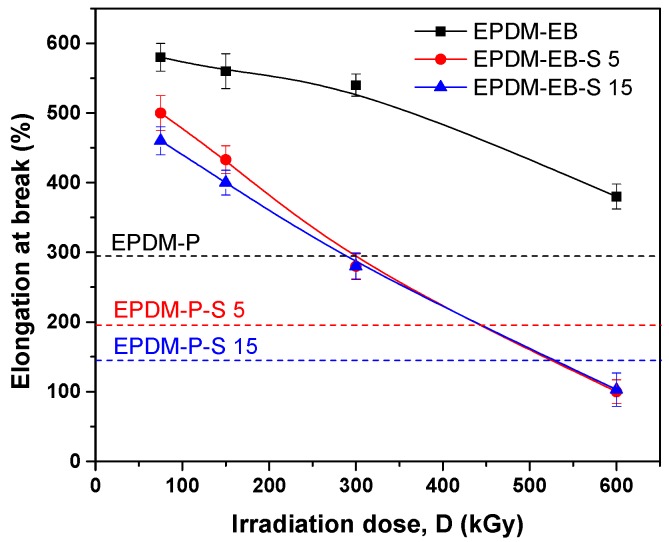
The influence of sawdust amount and cross-linking method (P/EB) on composites’ elongation at break.

As seen in Figure 4, irrespective of the irradiation dose below 300 kGy, the elongations at break of the composites containing S are superior to those measured for samples cross-linked by the P method. Differences between the samples containing the same S amounts and cross-linked by P/EB methods are more than 300%. The elongation at break decreasing with the increase of the irradiation dose and S amount indicates an increase in cross-link density. Furthermore, the addition of S can lead to the appearance of a restriction in the molecular chains’ movement [8,51]. Additionally, the striking forces between the filler and the polymer molecules led to the development of a cross-linked structure, which limit the free mobility of the polymer chains. Hence, this increases the resistance to accelerate upon the execution of tension [8,51,52]. The results are comparable with other obtained using different reinforcing fillers [1]. 

**Figure 5:**
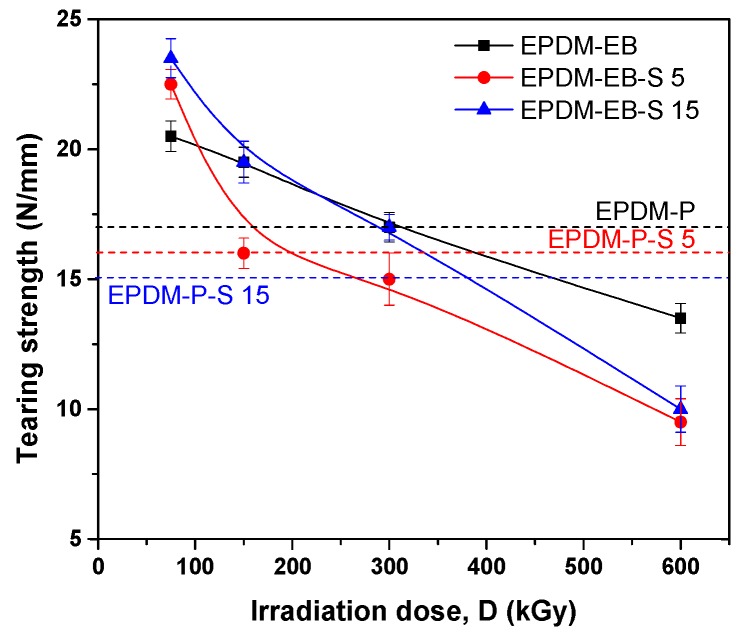
The influence of sawdust amount and cross-linking method (P/EB) on composites tearing strength.

Additionally, as above, in Figure 5, it can be seen that tearing strength of EPDM-EB and EPDM-EB-S 15 samples up to 300 kGy are over the values obtained in the case of P cross-linking with or without S. More than up to 150 kGy, tearing strength for samples EPDM-EB-S 15 is even over that of EPDM-EB samples. Considerable differences (up to 60%) can be observed between samples containing the same S amounts and cross-linked by P/EB methods.

The results presented above indicate that the EB irradiation and S addition have a reinforcing effect on EPDM rubber and conduct to special and different properties than in P cross-linking. This result can be explained by a different chemical nature of free radicals formed by radiation action that help for the addition to double bonds of unsaturated rubbers unlike those formed in P decomposition [3,53,54]. The relative reactivity or stability of the free radicals generated in P decomposition is related to the hydrogen bond dissociation energy of the parent compound [3,53]. Due to the high values of bond dissociation energy, methyl, phenyl, tert-butoxy, and other alkoxy radicals are highly reactive and are good hydrogen abstractors. Opposite ethyl, tert-butyl, and isopropyl radicals have lower values of bond dissociation energy. Therefore, they are poor hydrogen abstractors [53,55,56,57]. The reactivity of peroxide radicals depend not only on their structure but also on their size [3].

### 3.2. Gel Fraction and Cross-Link Densities

The cross-linking evaluation was done based on the gel fraction and cross-link density measurements. The results represent the average of five specimens.

**Figure 6:**
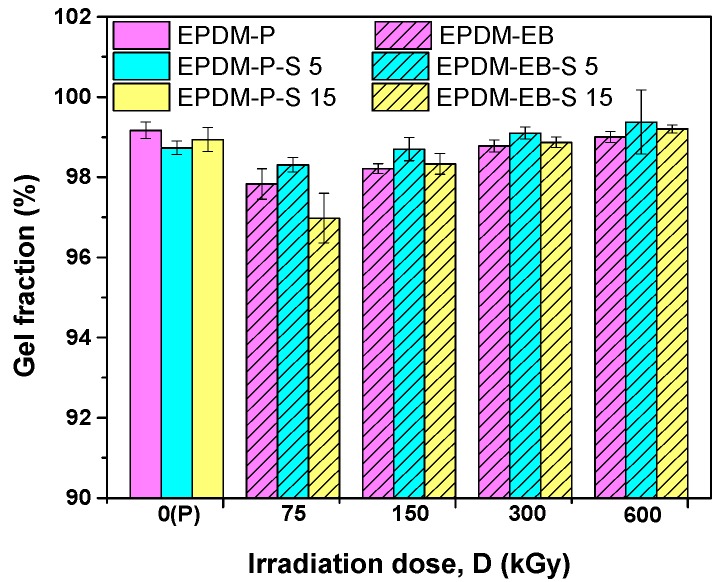
The effect of the cross-linking method (P/EB) and sawdust amount on the gel fraction.

As seen in Figure 6, gel fraction exhibit values over 98% (excepting for the EPDM-EB and EPDM-EB-S 15). 

As the irradiation dose increased, the gel fraction also increased. The results obtained at the highest irradiation dose of 300 and 600 kGy are similar with those obtained in the case of peroxide cross-linking. In addition, samples containing 5 phr S cross-linked with EB (EPDM-EB-S 5) exhibit higher gel fractions than those without S (EPDM-EB) and with 15 phr S (EPDM-EB-S 15). Thus, in the case of P cross-linking, it is necessary to add more S (15 phr) to obtain the same results as in the case of EB cross-linking (5 phr S/300 kGy). To obtain improved results, the sample was irradiated over 300 kGy (5 phr S/600 kGy).

**Figure 7:**
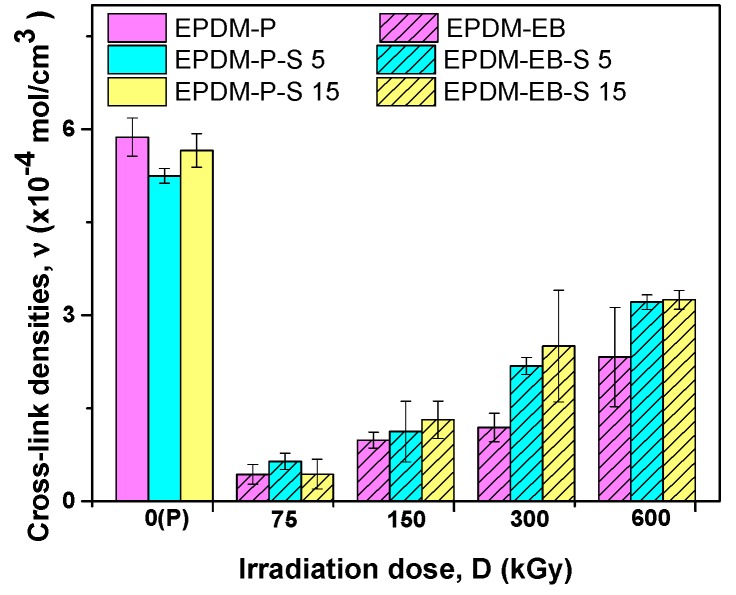
Effect of the cross-linking method (P/EB) and sawdust amount on the gel fraction.

The cross-linking process evaluation was also done by calculating cross-link density for P and EB cross-linked samples. The results are presented in Figure 7.

As seen in Figure 7, the cross-link densities of samples cross-linked by EB, with or without S, have increased with the irradiation dose increasing. In addition, for irradiation doses over 150 kGy, the cross-link density grows closely following the increase in filler load [47]. The increase of the network density could be explained by the composite rigidity increasing due to the S presence or by some specific chemical interactions between the S and EPDM matrix. The same behavior was observed in the case of hardness dependence on the S content [47]. 

Correlating the mechanical properties with the cross-linking evaluation, we can conclude that S acts similar to active fillers in both EPDM-P-S [47] and EPDM-EB-S composites and leads to the improvement of the composite properties [42,47,58,59,60,61]. Even in Figure 7, it can be observed that the P cross-linking led to the obtainment of better cross-link densities than EB cross-linking, by correlating the results with those presented in Figure 1, Figure 2, Figure 3, Figure 4 and Figure 5 up to 300 kGy. The results show that all mechanical properties of EPDM-EB and EPDM-EB-S samples were superior. This can be explained by stiffness and poor elasticity due to the lower mobility of the macromolecular chains at high cross-link densities [42,48,58,59,60,61]. 

### 3.3. FTIR Analysis

For a composite structure investigation (identification of different functional groups, presence or absence of specific functional groups), Fourier Transform Infrared (FTIR) spectroscopy was used [62]. 

**Figure 8:**
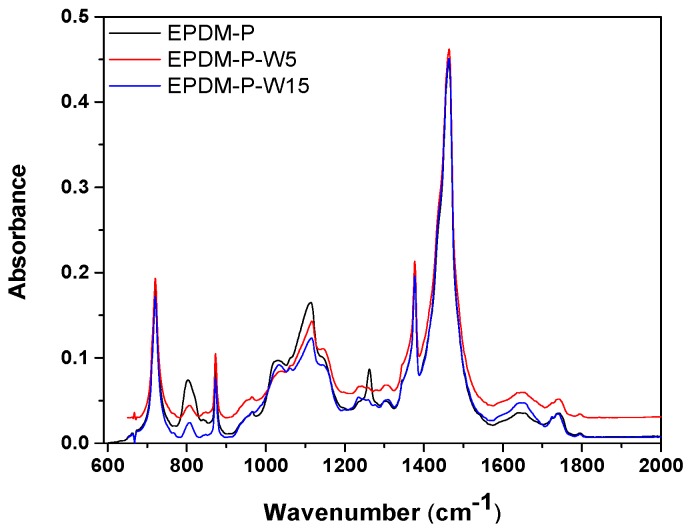
FTIR spectra of EPDM-S composites cross-linked by peroxide (P).

**Figure 9:**
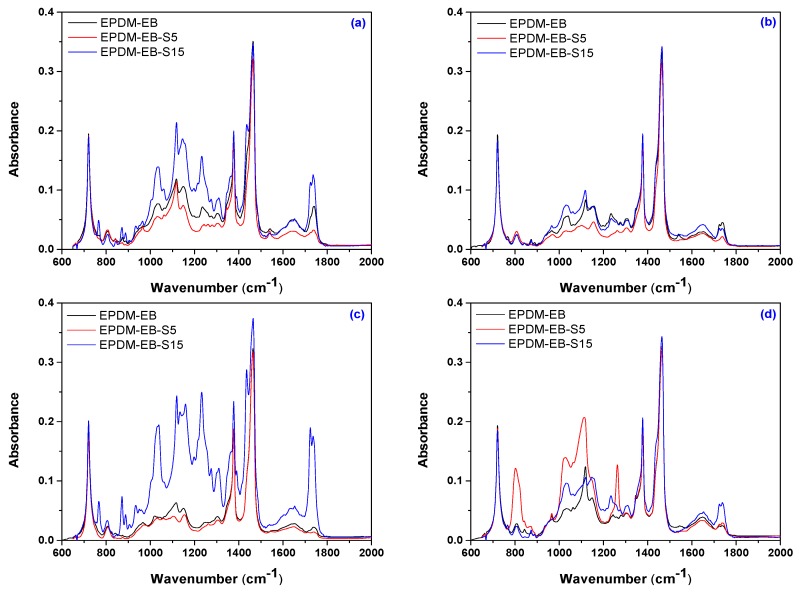
FTIR spectra of EPDM-EB-S composites cross-linked by EB irradiation at 75 kGy (**a**), 150 kGy (**b**), 300 kGy, (**c**) and 600 kGy (**d**) in the range of 650–2000 cm^−1^.

Figure 8 and Figure 9 present the infrared spectra obtained in the range of 2000–650 cm^−1^ on EPDM-P/EPDM-P-S and EPDM-EB/EPDM-EB-S composites due to the valence and deformation vibration of the atoms involved in the existing or formed covalent bonds. In addition, details of FTIR spectra in the range of 4000–3000 cm^−1^ on EPDM-EB-S 5 and EPDM-EB-S 15 composites will be presented. 

Due to the covalent bonding of the monomer units in EPDM rubber, they can be considered as being separate chemical components. Intra-chain (between adjacent monomer units in the polymer chain) and inter-chain (between monomer units that are not adjacent in the same polymer chain) interactions may be happening [63]. Due to the intra-chain interactions, ethylene and propylene groups are sensitive to the identities of adjacent groups in the polymer chain. On the other hand, polymer crystallinity and morphology are consequences of inter-chain interactions [63].

In the EPDM spectra, three absorption regions were identified as being significant. The first one was dominated by second overtone C–H stretching bands and located between 1100–1350 cm^−1^. The second one has a higher absorptivity with an order of magnitude, dominated by first-overtone C–H stretching bands, and located between 1570–1850 cm^−1^ and the third one that contains C–H combination bands with much higher absorptivity than those of the bands in regions one and two and located between 1950–2500 cm^−1^ [63].

**Figure 10:**
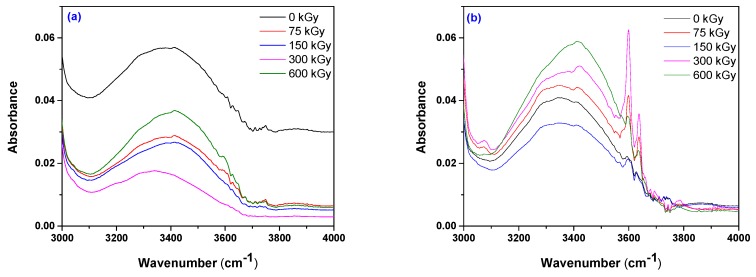
FTIR spectra of EPDM-EB-S 5 (**a**) and EPDM-EB-S 15 (**b**) composites cross-linked by EB irradiation.

The FTIR spectra of EPDM-P-S (Figure 8)/EPDM-EB-S (Figure 9 and Figure 10) show the presence of the EPDM specific bands, located in the above specified regions. 

Thus, this includes C–H stretching vibration (2918 and 2850 cm^−1^) [63,64,65,66,67], C=C stretching vibration (1630 cm^−1^) [68,69,70], CH_2_ bending and rocking vibrations (1460 and 720 cm^−1^) [64,65,66,67], and CH_3_ bending vibration (1376 cm^−1^) [64,65,66,67]. In Figure 8, all fingerprints of dibenzoyl peroxide were found including a weak absorption band in the region 950–800 cm^−1^ due to the O–O stretching vibration, a strong band at 1775 cm^−1^ (saturated aliphatic) due to the C=O stretching vibration, and a band between 1300–1050 cm^−1^ due to the C–O stretching vibrations emphasizing its domination over the O–O bond [71]. 

The main chemical components of wood sawdust are carbon (60.8%), hydrogen (5.2%), oxygen (33.8%), and nitrogen (0.9%) [72,73]. Cellulose (38%–50%) is the one that gives the wood stiffness [73,74]. Lignin (15–25%) is the cementing agent or resin in wood [73,75] and hemicelluloses (23–32%) are the bonding agent between cellulose and lignin [73]. The primary and secondary hydroxyls, carbonyls, carboxyls, esters, or ethers from cellulose, hemicelluloses, and lignin are examples of active functional groups that can be involved in chemical reactions [76,77,78,79].

The wood strength can be significantly reduced by EB irradiation [73] due to a decrease of polymerization degree and crystallinity and to the increase of the hydrolysis rate and yield of cellulose [76,77,78]. 

In Figure 8, Figure 9a–c, and Figure 10, the following basic structures of sawdust have been found in the obtained composites: a broad band between 3600–3100 cm^−1^ due to the OH-stretching vibration, which gives important information about the hydrogen bonds [80,81], a strong broad OH stretching (3598–3637 cm^−1^) that includes inter and intra-molecular hydrogen bond vibrations in cellulose [62], C–H stretching of all hydrocarbon constituents in polysaccharides [62] including methyl and methylene groups (2800–3000 cm^−1^), and a strong broad superposition with sharp and discrete absorptions in the region from 1000 to 1750 cm^−1^ [82]. 

Notable differences between EPDM-P, EPDM-P-S, and even between EPDM-P-S 5 and 15 in some regions can be observed in Figure 8. Thus, except the regions 1340–1550 cm^−1^ and 650–780 cm^−1^, all others absorb EPDM-P-S upper or under the EPDM-P with band intensities varying due to the possible degradation (chain scission/chain link cross-linking) processes and addition of the basic structure from S (especially between 1600–1800 cm^−1^ due to the ring stretching mode strongly associated with the aromatic C–O–CH_3_ stretching mode, the C=O stretching of conjugated/aromatic ketones, or the aromatic skeletal vibrations [82]) in EPDM-P-S 5 and EPDM-P-S 15 spectra [8,80,81]. Results can be correlated with those presented in Figure 7, where it is observed that the cross-link densities of the EPDM-P-S composites, especially EPDM-P-S 15, are comparable with those of EPDM-P. Thus, the use of other additive or fillers, as S, to replace sulphur (instead of increase the state of cure) are necessary additions to the cross-linking method (the cross-linking using only peroxide is low) [8,82].

By comparing the spectra presented in Figure 9 and Figure 10 with the spectra of cellulose, holocellulose, and lignin [82], the following specific bands have been found: aromatic skeletal vibrations caused by lignin (1510 and 1600 cm^−1^) and the absorption located at 1730 cm^−1^ caused by holocellulose. This indicates the C=O stretch in non-conjugated ketones, carbonyls, and in ester groups [82,83]. Appearance of the band near 1600 cm^−1^ is a relative pure ring stretching mode associated with the aromatic C–O–CH_3_ stretching mode [82]. The C=O stretch of conjugated or aromatic ketones absorbs below 1700 cm^−1^ [82] and can be seen as shoulders in the spectra.

Formation of active functional groups able to be involved in chemical reactions and radicals during the EB irradiation is responsible for the bonding process between the EPDM rubber matrix and filler (sawdust) [8,79].

The addition of 5 phr of S (Figure 10a) decreases the 3200–3600 cm^−1^ broad band intensity from 0.058 (for un-irradiated EPDM-S 5) to 0.035 (for EPDM-S 5 irradiated at 600 kGy), 0.029 (for EPDM-EB-S 5 irradiated at 75 kGy), 0.0275 (for EPDM-EB-S 5 irradiated at 150 kGy), and 0.018 (for EPDM-EB-S 5 irradiated at 300 kGy)—shifted band. The addition of 15 phr of S (Figure 10b) places from the beginning the intensity of the broad band at 3200–3600 cm^−1^ corresponding to un-irradiated EPDM-S under all other EPDM-EB-S 15 irradiated, except 150 kGy. 

The band assignments in EPDM-P-S and EPDM-EB-S samples from the FTIR spectra presented above are listed in Table 1.

The previously mentioned presence of wood components (cellulose, hemicellulose, and lignin) in EPDM-EB-S samples confirms their origin from the sawdust used for obtaining composites (Table 1). In Figure 10a,b, notable differences in band widths and intensities can be observed due to the cross-linking method and S loading. Thus, in the case of 5 phr S loading, all EB irradiation results are under P curing (Figure 10a). In the case of 15 phr S loading, except for the 150 kGy EB irradiation, all other absorbances were over the P curing absorbance and close to degradation. The results are very well correlated with the mechanical test results (Figure 1, Figure 2, Figure 3, Figure 4 and Figure 5) where EB irradiation with 150 kGy turned out to be the most effective dose for obtaining better effects than P curing.

### 3.4. Water Uptake Test Results

**Figure 11:**
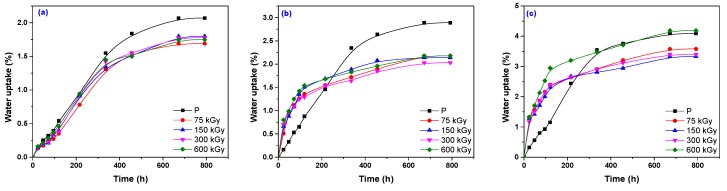
EPDM (**a**), EPDM-S 5 (**b**), and EPDM-S 15 (**c**) water uptake behavior as a function of immersion time.

The results of water uptake experiments that were carried out on samples with/without sawdust and treated by means of P and EB are presented comparatively in Figure 11. The cross-linking method (EB and P), S loading (5 and 15 phr), and irradiation dose (75, 150, 300, and 600 kGy) are responsible for composite absorption behavior. 

In Figure 11, it can be observed that water uptake equilibrium was reached earlier by the samples containing S, except in the case of EPDM-S 5 cured with P (Figure 11b). In addition, as the S loading increased, the water uptake increased from up to 2% for EPDM-EB-S 5 (Figure 11b) to up to 4% for EPDM-EB-S 15 (Figure 11c). Samples with or without S and cured by EB irradiation present absorptions lower than those cured by P (Figure 11a,b), except for the sample EPDM-EB-S 15 irradiated at 600 kGy (Figure 11c). The increase of water absorption with the S loading may be explained by both a hydrophilic nature of S and a big interfacial area between the S and the elastomer matrix [8,87]. In rubber composites that contain wood, water is absorbed mainly by the last one because, since rubbers are hydrophobic, their absorbability can be neglected [8,87]. The amount of free –OH groups from cellulose and hemicellulose increases when the S content increases. Their contact with water leads to the formation of hydrogen bonds and, consequently, the weight of the composite increases [8,88]. A reduced amount of –OH groups inside the composite may conduct to a low availability to absorb water [8,88].

There are several studies that have shown that P prevents the aggregation of filler in the rubber matrix forming a network structure and, in the same time, acts as a plasticizer for rubber and as a compatibilizer between the hydrophobic rubber and the hydrophilic filler. These properties are responsible for the improved interaction at the interface between EPDM rubber and sawdust [8,89,90,91].

### 3.5. Rubber-Filler Interaction

The Kraus equation was used to analyze the interaction between EPDM rubber matrix and the natural filler, which is sawdust. The results are listed in Table 2 in terms of volume fraction of rubber in the swollen gel (*V_rf_*) and degree of restriction of the rubber matrix swelling due to the presence of filler (*V_ro_/V_rf_* ratio) [8,92,93]. 

As shown in Table 2, the addition of a higher quantity of sawdust (15 phr) led to a slowly decreasing *V_ro_/V_rf_* ratio, except for the composites obtained at 150 kGy and 300 kGy. According to Kraus theory, reduced values of the *V_ro_/V_rf_* ratio are associated with a good adhesion between rubber and filler and with the appearance of the reinforcement effect [8,92,93]. 

Even if fillers generally reduce the swelling, high or enhanced values *V_rf_* depend on the density of the cross-links and are associated with complex networks that present a lower degree of swelling [8,92,93]. In addition, in Table 2, it can be seen that, for the same irradiation dose of 150 kGy and 300 kGy, the increase of the sawdust amount led to the *V_rf_* growing.

**Figure 12:**
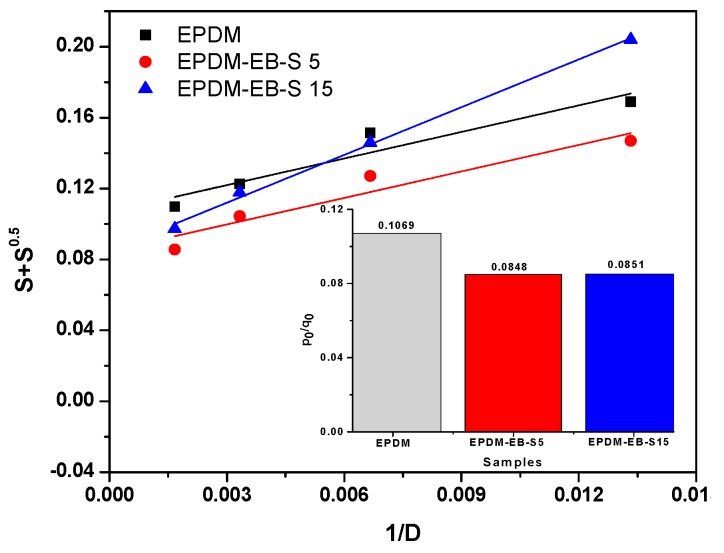
Plot of *S* + $\sqrt{S}$ vs. 1/absorbed dose (*D*) for EPDM-EB-S composites.

The quantitative evaluation of yields of cross-linking and chain scission of the EPDM rubber and EPDM-EB-S composites was done from the plots of *S* + $\sqrt{S}$ vs. 1/absorbed dose (*D*) from the Charlesby-Pinner equation for blend compositions (Figure 12) [8,94,95].

(2) $$S+\sqrt{S}=\frac{p_{0}}{q_{0}}+\frac{1}{\alpha P_{n}D}$$

where *S* is the sol fraction (*S* = 1-gel fraction), $p_{0}$ is the degradation density, average number of main chain scissions per monomer unit and per unit dose, $q_{0}$ is the cross-linking density, proportion of monomer units cross-linked per unit dose, *P*_*n*_ is the number averaged degree of polymerization, and *D* is the radiation dose in Gy.

In Figure 12, it is observed that the EPDM-EB-S 15 sample is the most effective cross-linked by EB irradiation. The cross-linking extent increases linearly with the S content. Low values of $p_{0}$/$q_{0}$ for high S content are suggestive for the relatively improved radical-radical interactions in the polymer composite [8,94,96,97]. Values under the unit of the $p_{0}$/$q_{0}$ ratio (0.1069 for EPDM, 0.0848 for EPDM-EB-S 5 and 0.0851 for EPDM-EB-S 15) indicate that the cross-linking prevailed over degradation.

## 4. Conclusions

Two classes of eco-composites based on ethylene-propylene-terpolymer rubber (EPDM) and wood sawdust (S) were obtained by two different methods: dibenzoyl peroxide cross-linking (P) at a high temperature and EB cross-linking at room temperature of 25 °C using the irradiation dose between 75 kGy and 600 kGy. 5 phr and 15 phr were the amounts of S used as filler. Even if both cross-linking methods insured gel fractions over 98%, the cross-link densities determined on samples cross-linked by the P method were higher when compared to those determined on samples cross-linked by EB. However, the addition of S increases the cross-link density of the composite in the case of the EB cure. Over the irradiation dose of 300 kGy, the composite becomes harder and less elastic, even for 5 phr S. The addition of 15 phr S to the samples irradiated over 300 kGy degrades the tensile properties of the composite. These results correlated with FTIR analysis show that the irradiation dose up to 300 kGy appears to be sufficient to obtain the reinforcement effect in the composite. After that, the process is susceptible to be close to degradation. FTIR analysis showed the cellulose radical formation during the irradiation and also the formation of active functional groups susceptible to chemical reactions that can be associated with the bonding process between the matrix (EPDM rubber) and the filler (S). The water uptake tests have shown very good absorption properties, especially for 5 phr S, irrespective of the irradiation dose.

The sawdust adding was carried out with the purpose of increasing the composite degradation potential without affecting its mechanical properties, as a solution for replacing the materials based exclusively on EPDM.

## Figures and Tables

**Table 1:** Band assignments in EPDM-P-S and EPDM-EB-S samples.

Band Position in the EPDM-P-S/EPDM-EB-S Composites (cm^−1^)	Functional Group
3360–3390, 3598, 3636, 3637 (EPDM-EB-S 15)	O–H stretching vibration (3300–4000 cm^−1^) from wood sawdust/cellulose [62]
2918	C–H stretching vibration (2800–3000 cm^−1^) from EPDM [64,65,66,67]
2850 (EPDM-P-S, EPDM-EB-S)	C–H stretching vibration (2800–3000 cm^−1^) from EPDM [64,65,66,67] and wood sawdust (polysaccharides/cellulose) [62]
1775 cm^−1^ (EPDM-P-S)	C=O stretching vibration in P [71]
1730–1740 (EPDM-P-S, EPDM-EB-S)	Aromatic skeletal vibrations caused by holocellulose (wood sawdust) [82] C=O stretch in non-conjugated ketones, carbonyls, and ester groups [82,83]
1640	C=C stretching vibration (1630 cm^−1^) from EPDM [68,69,70]
1642–1646 (EPDM-EB-S)	Typical bands assigned to cellulose—Vibration of water molecules absorbed in cellulose [62]
1539, 1540 (EPDM-P-S)	Aromatic skeletal vibrations caused by lignin (wood sawdust) [82] C=O stretch in non-conjugated ketones, carbonyls, and in ester groups [82,83]
1460	CH_2_ bending and rocking vibrations from EPDM [64,65,66,67]
1435, 1436 (EPDM-EB-S)	Stretching and bending vibrations of –CH_2_ bonds, associated with the amount of the crystalline structure of the cellulose [62,84,85]
1376	CH_3_ bending vibration from EPDM [64,65,66,67]
1034 (EPDM-EB-S)	Stretching and bending vibrations of –OH bonds in cellulose [62,84,85]
1300–1050 cm^−1^ (EPDM-P-S)	C–O stretching vibrations emphasizing its domination over the O–O bond in P [71]
950–800 cm^−1^ (EPDM-P-S)	O–O stretching vibration in P [71]
930 (EPDM-EB-S)	Assigned to the amorphous region in cellulose [62,86]
905 (EPDM-EB-S)	C–O bonds in cellulose [40,84,85]
720	CH_2_ bending and rocking vibrations from EPDM [64,65,66,67]

**Table 2:** *V_rf_* and *V_ro_*/*V_rf_* of EPDM-S composites determined in toluene.

Irradiation Dose	Samples	*V_rf_*	*V_ro_/V_rf_*
75 kGy	EPDM-EB-S 5	0.1912	0.8609
	EPDM-EB-S 15	0.1516	1.0862
150 kGy	EPDM-EB-S 5	0.2920	0.7787
	EPDM-EB-S 15	0.2964	0.7670
300 kGy	EPDM-EB-S 5	0.2242	1.0760
	EPDM-EB-S 15	0.2358	1.0232
600 kGy	EPDM-EB-S 5	0.3401	0.8922
	EPDM-EB-S 15	0.3202	0.9478
